# Development and psychometric properties of the Suicidality: Treatment Occurring in Paediatrics (STOP) Suicidality Assessment Scale (STOP-SAS) in children and adolescents

**DOI:** 10.1186/s12887-016-0751-2

**Published:** 2016-12-13

**Authors:** I. Flamarique, P. Santosh, A. Zuddas, C. Arango, D. Purper-Ouakil, P. J. Hoekstra, D. Coghill, U. Schulze, R. W. Dittmann, J. K. Buitelaar, K. Lievesley, R. Frongia, C. Llorente, I. Méndez, R. Sala, F. Fiori, J. Castro-Fornieles, the STOP consortium, A. Sutcliffe, Sarah Curran, Laura Selema, Matthew Hollocks, Ewa Nowotny, Robert Flanagan, Ian Craig, Nathan Parnell, Keren Yeboah, Jatinder Singh, Florence Pupier, Loes Vinkenvleugel, Jeffrey Glennon, Mireille Bakker, Cora Drent, Elly Bloem, Mark-Peter Steenhuis, Ruth Berg, Alexander Häge, Mahmud Ben Dau, Konstantin Mechler, Sylke Rauscher, Sonja Aslan, Simon Schlanser, Ferdinand Keller, Alexander Schneider, Paul Plener, Jörg Fegert, Jacqui Paton, Macey Murray, Noha Iessa, Sara Bahadori, Claire Baillon, Hugo Peyre, David Cohen, Olivier Bonnot, Julie Brunelle, Nathalie Franc, Pierre Raysse, Véronique Humbertclaude, Ana Espliego, Jessica Merchán, Cecilia Tapia, Lara Kehrmann, Immaculada Baeza, Soledad Romero, Amalia La Fuente, Ana Ortiz, Helen Furse, Nick Penkov, Alfred C. Kolozsvari, Corina Bodea, Manuela Pintor, Franca Ligas, Francesca Micol Cera, Bruno Falissard, Ameli Schwalber, Juliane Dittrich, Andrea Wohner, Katrin Zimmermann, Andrea Schwalber, Katherine Aitchison

**Affiliations:** 1 Department of Child and Adolescent Psychiatry and Psychology, SGR1119, Institut Clinic de Neurociències, Hospital Clínic Universitari of Barcelona, Fundació Clínic per la Recerca Biomèdica, C/Villarroel, 170, Barcelona, 08036 Spain; 2 Centro de Investigación Biomédica en Red de Salud Mental, CIBERSAM, Madrid, Spain; 3 Department of Child and Adolescent Psychiatry, Institute of Psychiatry, Psychology and Neuroscience (IoPPN), King’s College London, London, UK; 4 Centre for Interventional Paediatric Psychopharmacology, South London and Maudsley NHS Foundation Trust, London, UK; 5 Department of Biomedical Sciences, Cagliari University Hospital, University of Cagliari, Cagliari, Italy; 6 Department of Child and Adolescent Psychiatry, Hospital General Universitario Gregorio Marañón, CIBERSAM, IiSGM, School of Medicine, Universidad Complutense de Madrid, Madrid, Spain; 7 CHRU Montpellier, Hôpital Saint Eloi, Médecine Psychologique de l’Enfant et de l’Adolescent, Montpellier, France; 8 INSERM U894-Team 1. Center of Psychiatry and Neurosciences, Paris, France; 9 Department of Psychiatry, University of Groningen, University Medical Center Groningen, Groningen, The Netherlands; 10 University of Dundee, Dundee, UK; 11 Department of Child and Adolescent Psychiatry/Psychotherapy, University of Ulm, Ulm, Germany; 12 Paediatric Psychopharmacology, Department of Child and Adolescent Psychiatry, Central Institute of Mental Health (CIMH), Medical Faculty Mannheim, University of Heidelberg, Mannheim, Germany; 13 Department of Cognitive Neuroscience, Donders Institute for Brain, Cognition and Behavior, Radboud University Medical Centre, and Karakter Child and Adolescent Psychiatry University Centre, Nijmegen, The Netherlands; 14 Department of Psychiatry and Clinical Psychology, University of Barcelona, Barcelona, Spain; 15 Institut d’Investigació Biomèdica August Pi i Sunyer, IDIBAPS, Barcelona, Spain

**Keywords:** Suicidality, Adverse events, Adolescents, Children, Parents, Assessment, Scale

## Abstract

**Background:**

To create a self-reported, internet-based questionnaire for the assessment of suicide risk in children and adolescents.

**Methods:**

As part of the EU project ‘Suicidality: Treatment Occurring in Paediatrics’ (STOP project), we developed web-based Patient Reported Outcome Measures (PROMs) for children and adolescents and for proxy reports by parents and clinicians in order to assess suicidality. Based on a literature review, expert panels and focus groups of patients, we developed the items of the STOP Suicidality Assessment Scale (STOP-SAS) in Spanish and English, translated it into four more languages, and optimized it for web-based presentation using the HealthTracker^TM^ platform. Of the total 19 questions developed for the STOP-SAS, four questions that assess low-level suicidality were identified as screening questions (three of them for use with children, and all four for use with adolescents, parents and clinicians). A total of 395 adolescents, 110 children, 637 parents and 716 clinicians completed the questionnaire using the HealthTracker^TM^, allowing us to evaluate the internal consistency and convergent validity of the STOP-SAS with the clinician-rated Columbia Suicide Severity Rating Scale (C-SSRS). Validity was also assessed with the receiver operating characteristic (ROC) area of the STOP-SAS with the C-SSRS.

**Results:**

The STOP-SAS comprises 19 items in its adolescent, parent, and clinician versions, and 14 items in its children’s version. Good internal consistency was found for adolescents (Cronbach’s alpha: 0.965), children (Cronbach’s alpha: 0.922), parents (Cronbach’s alpha: 0.951) and clinicians (Cronbach’s alpha: 0.955) versions. A strong correlation was found between the STOP-SAS and the C-SSRS for adolescents (r:0.670), parents (r:0.548), clinicians (r:0.863) and children (r:0.654). The ROC area was good for clinicians’ (0.917), adolescents’ (0.834) and parents’ (0.756) versions but only fair (0.683) for children’s version.

**Conclusions:**

The STOP-SAS is a comprehensive, web-based PROM developed on the HealthTracker^TM^ platform, and co-designed for use by adolescents, children, parents and clinicians. It allows the evaluation of aspects of suicidality and shows good reliability and validity.

## Background

According to the World Health Organization, approximately one million people commit suicide each year worldwide [1]. The age range with the greatest risk of suicidal behaviour is adolescence and early adulthood [2, 3]. Lifetime prevalence rates in adolescents are reported cross-nationally to be 19.8–24.0% for suicidal ideation and 3.1–8.8% for suicide attempts [4].

Suicidality, however, is a complex behaviour with many interrelated elements. It comprises the following elements: suicidal ideation (thoughts about death, a wish to be dead), suicide plans (thinking about ways of carrying out a self-injurious behaviour that could result in death), suicide communication or threats (transmitting or expressing thoughts about or the intention of suicide, either explicit or implicit), suicide behaviours involving self-harm (self-injuries with no intent to die), and suicide attempts (self-inflicted, dangerous behaviour with the aim of dying but a non-fatal outcome) [4, 5].

The term ‘Medication-Related Suicidality’ (MRS) refers to any suicide-related symptoms that are reported during the period of treatment with a drug. In particular, previous studies have shown a weak but significant risk of self-harm and suicidal ideation in adolescents treated with antidepressants [6, 7]. As a consequence, the assessment of suicidality in both pre- and post-marketing clinical trials of the various drugs developed by pharmaceutical companies has become a pressing issue. Other instruments that evaluate suicidality include the Suicidal Ideation Questionnaire Junior [8], the Suicidal Behaviours Questionnaire-Revised [9], the Sheehan Suicidality Tracking Scale [10] and the Beck Suicide Ideation Scale [11] among others. A key distinction between these existing measures is that the majority of previous measures have been developed for use in adult patients and almost exclusively have not been developed for web-based completion. Most of them have been developed for adult patients and are not web-based questionnaires. There is a need to develop measures to specifically assess suicidality in the child and adolescent populations given the specific stresses they experience at this age. Since suicidality remains a delicate topic, it can be argued that a proven web-based health monitoring platform could be particularly useful in this population, as they provide a space for honest responses to sensitive topics.

Patient Reported Outcome Measures (PROMs) have enabled patients to be more forthcoming in disclosing information about subjective states of health to a clinician, including suicidal ideation and behaviour [12]. PROMs include self/proxy assessments of outcomes and experience of symptoms, functional status, patient needs, and satisfaction with care [13–15].

The STOP project (Suicidality: Treatment Occurring in Paediatrics; grant agreement number: 261411) is a response to a specific research call made under the FP7 Cooperation Work Program ‘HEALTH.2010.4.2-3: Adverse drug reaction research’. The aim of the STOP project was to develop and validate a comprehensive web-based module for the assessment and monitoring of suicidality and its mediators in children and adolescents using the HealthTracker™ health monitoring platform.

In this study, which is part of the STOP project, we describe the development of a self-reported, web-based questionnaire for assessing suicide risk in children and adolescents, which aimed specifically to capture the different elements of suicidality. The instrument, known as the STOP Suicidality Assessment Scale (STOP-SAS), consists of two self-report questionnaires (one for children aged 8–11 years, one for adolescents 12–18 years) and two parent-report and clinician-report questionnaires. The questionnaires were administered to a sample of adolescents, children, parents and clinicians and validated against the C-SSRS. A secondary aim of the present study was to identify a set of questions from the STOP-SAS that assess low-level suicidality that could be used for screening.

## Methods

### STOP Project

The checklist was developed as part of the EU FP7 project—“Suicidality: Treatment Occurring in Paediatrics (STOP study; www.stop-study.com)” [16]. The STOP project aimed to develop a comprehensive web-based methodology for the assessment and monitoring of suicidality in children and adolescents. This study focussed on developing valid web-based suicidality measures (in multiple languages) for children and adolescents on the HealthTracker^TM^ (a health monitoring platform), for use in assessing suicidality, detecting impact of medication on suicidality, and suicidality related risk and resilience factors. The participants, parents and clinicians completed a wide-ranging battery of scales in order to report the symptom experience (both related to suicidality and symptoms generally), medication adherence and compliance, and quality of life. This manuscript describes specifically the development of the STOP-SAS.

### HealthTracker^TM^

HealthTracker^TM^ (https://www.healthtracker.co.uk), a multi-modal real time web-based e-health monitoring tool, is suitable for risk stratification and monitoring and post-marketing surveillance of medication as it can capture longitudinal data about behaviour, emotions, side-effects, cognitive functions, quality-of-life of both the child/adolescent and family along with details of all medication used as well as medication adherence and compliance [17]. The HealthTracker^TM^ optimises clinician time by helping to profile young people before being seen in clinic and is used to monitor patient-centred outcomes as part of clinical treatment.

### Ethical Approval

All centres had ethical approval for the study from their local committees using the standardised protocol. The UK (London) was the lead site for the STOP Project and the ethics details were: REC Reference Number 13/LO/0401; Kent Research Ethics Committee and Institute of Psychiatry, King’s College London Research and Development Office.

### Item Generation and Preliminary Scale Development

The development of the instruments followed the FDA recommendations for Patient Reported Outcome Measures (PROMS) [15]. The initial items of the STOP-SAS were developed via consultation of respective publications in the literature and existing scales, and by considering the aspects assessed by the C-SSRS [18]. The schema developed by Silverman [19], which differentiates between the presence or absence of suicidal intent, and between the presence or absence of injury, was also considered [15]. Suicidal behaviours with no intent to die include those in which the person wishes to use the appearance of killing him/herself for other purposes (e.g., seeking help, punishing others, receiving attention, regulating negative mood) while suicidal behaviours involving an undetermined intent to die are those in which the intention is unknown (e.g., unconsciousness, dissociation, being under the influence of drugs, being delusional, or reluctance to admit to the intent to die) [19]. Other scales such as the Suicidal Ideation Questionnaire (Junior High School Version) [8], as well as expert opinions were also taken into consideration in order to develop the first version of the scale. The first version of the STOP-SAS, which included 12 items, was developed simultaneously in English and Spanish and was discussed thoroughly with a panel of European experts from Spain, UK, France, Italy and Germany.

### Focus Groups

Patients’ understanding of the initial STOP-SAS scale was assessed through cognitive interviewing in eight focus groups with children, adolescents and parents. This qualitative research tool was used to ensure understanding of the concepts contained in the items. Groups were small, consisting of three or four individuals recruited at the outpatient unit of the Child and Adolescent Psychiatry Department of the Hospital Clinic of Barcelona. Participants and their parents completed an assent and consent form. The Hospital’s Ethics Committee approved the study. With the permission of participants, all the group sessions were video-recorded. Participants were asked a combination of standard probes and on-the-spot probes (verbal probing method) [20]. After each focus group the content of the videotapes was transcribed. The transcripts were analyzed using the thematic analyses and cut-and-paste method, and a detailed summary report was written regarding each focus group [21, 22]. Discussions between experts from UK and Spain were held to produce the final version of the questionnaire. A clinician’s version was also created based on the parents’ and adolescents’ versions.

**Fig. 1:**
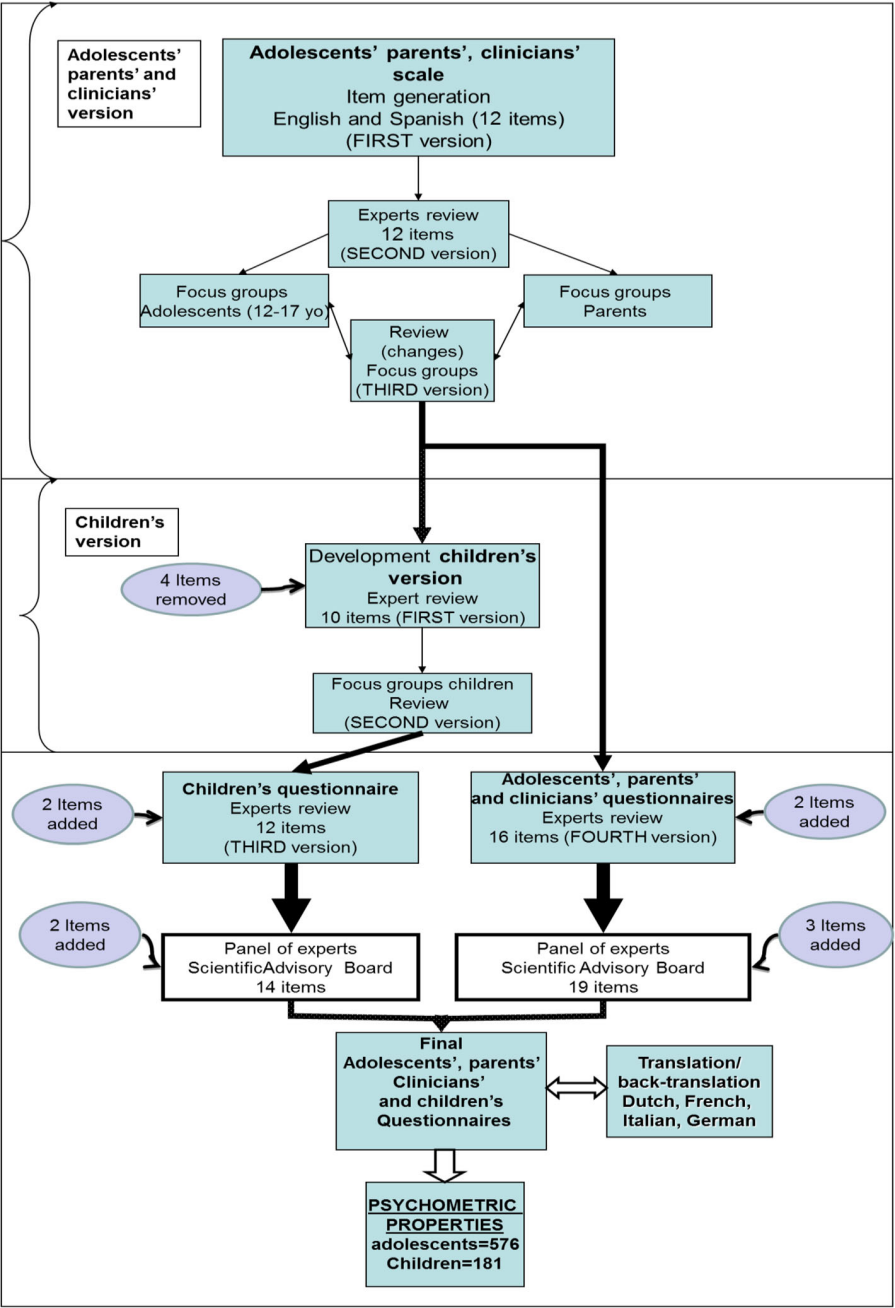
Flow chart summarizing the development of the scales

The final versions of the questionnaires in English and Spanish were reviewed by a professional translator for comparability of the questions in the two languages. The STOP scientific advisory board also suggested some changes that were implemented by the expert panel and authorized by the consortium. The English scales were then translated into German, Dutch, French and Italian, and then back-translated into English. Discussions between experts from each country were held to ensure that the meaning of each statement remained culturally sensitive, and the questionnaires were then finalized. A flow chart of the creation of the STOP-SAS is shown in Fig. 1. The definitive questionnaires were then uploaded onto HealthTracker™, an online multimedia platform with a suite of questionnaires for monitoring health that allow accurate measurements of change across a wide range of symptoms, adverse events, psychological functions and quality of life. It was developed as a multi-informant system for use by children, adolescents, parents, teachers, clinicians and researchers and allows for questionnaires to be presented in a user-friendly manner, assisted by audio recordings for those with hearing problems [16, 17]. The system also allows the clinician to assign the questionnaires taking into consideration the mental age of the respondent. This flexibility is important, especially for those with intellectual or learning disabilities. For severely impaired children, one must rely on parent and clinician proxy questionnaires [17].

### Psychometric properties of the scale

To carry out the evaluation of the STOP-SAS’s psychometric properties (internal consistency and convergent validity), the questionnaires were completed online via the HealthTracker™ system by a sample of patients (adolescents and children), their parents and clinicians from the various participating centres. These centres were specifically selected as part of a consortium based on expert child and adolescent psychiatric practice. There were 2 sites in Spain (Madrid and Barcelona), 2 sites in the UK (London and Dundee), one site in Italy (Cagliari), 3 sites in France (2 hospitals in Paris and one in Montpellier), 2 sites in Germany (Ulm and Mannheim) and 2 further sites in the Netherlands (Groningen and Nijmegen). The clinicians completing the questionnaires were the expert psychiatrists who worked with the young person in the clinical setting.

A total of 763 parents/legal tutors of patients from the different centres signed an informed consent to participate in the study. Of these 763 families, 576 included an adolescent (age between 12 and 17 years old), 187 a child (age between 8 to 11 years old). In some of the cases only parents or clinicians answered the questionnaires due to intellectual or learning disabilities of the patient. For the assessment of convergent validity, the C-SSRS was used. Validity was also assessed using the receiver operating characteristic (ROC) analyses. For this purpose we used the gold standard (C-SSRS) total score and created a dichotomous variable: if the total score was other than “zero” the value was “1” (suicidality is present) and it was “zero” the value was “0”(suicidality is not present). The C-SSRS is a clinician-administered questionnaire that assesses suicidal ideation and suicidal behaviours based on several ordinal items. Suicidal behaviours include suicide attempts and completed suicides, as well as any preparatory act with a view to attempting suicide [23].

### Screening questions

Since the investigators, the Scientific Advisory Board, and Ethics Committees recommended reducing the burden of answering a large number of questions regarding suicidality in non-suicidal subjects, the experts then identified a set of four questions from the STOP-SAS that could be used as screening questions (three of them for use with children, and all four for use with adolescents, parents and clinicians). These items were selected because they assess low-level suicidality. Using data from a subsample of patients who responded to all the questions, we checked that if a patient or parent answered “never” to all the screening questions they also answered “never” to all the remaining questions of the STOP-SAS. This subsample comprised a total of 53 children, 93 adolescents and their parents and clinicians who answered all the questions. A negative answer to all the screening questions would then be considered as a total score of “zero” for the entire STOP-SAS, while one positive answer to any of these questions would automatically require the participant to complete the entire questionnaire. Experts ensured that the set of four screening questions for adolescents, parents and clinicians, and the three for use by children fulfilled this criterion.

### Data analysis

#### Descriptive Statistics

Descriptive statistics (percentages, means and standard deviations) were used to summarize the results.

#### Psychometric Properties of the STOP-SAS

A one-way between-groups multivariate analysis of variance was performed to compare the mean total scores between groups (parents, clinicians and children or adolescents). A total score was calculated by adding up the values of the response options (0 to 5 for adolescents, parents and clinicians and from 0 to 3 for children). The total scores were then transformed to a range from 0 to 100, so children’s scores were comparable to parents’ and clinicians’. The Mauchly’s Test of Sphericity was used to evaluate Sphericity. The Greenhouse-Geisser correction was used if the assumption of sphericity had been violated. A *t*-test was then applied for post-hoc analysis (*t*-test with Bonferroni correction for multiple comparisons). Internal consistency was estimated using Cronbach’s alpha coefficient. Pearson’s correlation was used to assess correlation between respondents and convergent validity between the STOP-SAS and the C-SSRS. A correlation coefficient of >/= .10 represents a weak association, one of >/= .30 a moderate correlation, and one of >/= .50 or larger a strong correlation [24]. Validity was also assessed using the receiver operating characteristic (ROC) analyses using the C-SSRS as the gold standard. Cut off scores, sensitivity, and specificity were also computed. All statistical analyses were performed using the SPSS20.0 software package.

#### Screening questions analyses

In order to statistically test the predictive power of the screening questions on the overall score we created two variables: one was obtained by the sum of the first four items scores (Or three for the children’s version) and the other was the sum of the scores of all the rest of the questions. Pearson’s correlation was used to assess correlation between these two new variables. Moreover, these two variables were transformed in two dichotomous variables called “Screening-questions” and the “STOP-SAS-RestQ”. These variables might have two values: “0” if the sum of all the items which compose the variable is zero and “1” for all the other possible scores. We calculated the Kappa measure of agreement between the “Screening-questions” and “STOP-SAS-RestQ”.

## Results

### Focus Groups

- *Adolescents’, parents’ and clinicians’ questionnaires*

Three focus groups were created, each comprising three or four adolescents aged between 12 and 17 years old (total *n* = 10). Sixty per cent of the total sample was female, and the primary diagnoses were anorexia nervosa (*n* = 5), major depression (*n* = 3) and anxiety disorders (*n* = 2). The mean age of the sample was 15.60 ± 1.07 years. The focus group sessions lasted between 70 and 85 min. Overall, adolescents expressed their preference for a computer-based scale rather than a printed version. The recall period suggested by participants was 1 month. They also suggested that some items from the STOP-SAS should be re-worded, although none needed to be removed. They thought that being asked about suicide was important and necessary for suicide prevention. A number of different response scales were tested (including 7-point and 6-point Likert scales) in order to determine the optimal number of response options. Participants preferred six options.

Five parents of the adolescents also participated in two focus groups for parents. They understood all the items included in the instrument. They suggested that two questions which included concepts about “hurting yourself” and “killing yourself” in the same item should be presented separately, and so two new items were added.

The third version of the instrument for adolescents, parents and clinicians thus consisted of 14 items.

- *Children’s questionnaire*

The first version of the questionnaire for children between 8 and 11 years was a simplified version of the version for adolescents. Four items were removed, and it was decided to use a 4-point Likert-type response. Some items and the response options were re-worded using more age-appropriate vocabulary, while other statements were shortened. The first version thus consisted of ten questions. This version was presented to the three focus groups comprising three children each, aged between 8 and 11 years. The focus groups lasted between 30 and 55 min. The children understood all questions, but some of them were re-worded as children had some difficulties in differentiating them. They also suggested including some examples to make them easier to understand. The recall period was modified for children, with ‘1 month’ being changed to ‘over the last few days’. The second version of the children’s instrument also consisted of ten items.

### Final agreement on scale formats between experts

- *STOP-SAS Adolescent’, Parent’ and Clinician’ versions*

The revised adolescent version was reviewed by the UK and Spanish experts, who agreed to add a further two items about suicide plans, namely ‘preparatory acts’ and ‘reasons’ in order to include all the components of suicidality and to map the C-CASA algorithm. The resulting versions designed for adolescents, parents and clinicians had five items referring to suicidal ideation, six items to suicidal behaviour, four to suicide plans and one to suicide communication. On the advice of the STOP Scientific Advisory Board, we added three items referring to suicidal ideation, as they felt that the scale required some questions assessing low-level suicidality. Thus, the final versions for adolescents, parents and clinicians consisted of 19 questions. All three versions included the same items and the same content, with a slight change in the questions depending on the respondent to make it clear that the questions were about the child/adolescent. The panel of experts decided to add a stand-alone item to ask about potential lethality to the clinician’s version. This question was only visible if there was a positive answer to behaviour and there were no injuries associated to that behaviour. This item was not considered a part of the questionnaire and it will not be considered in the analysis. Table 1 shows the different items that comprise the instrument.

**Table 1:** Items of the different versions of the STOP-SAS instrument

Item Name	Adolescents’ version	Parents’ version	Clinicians’ version	Children’s version
Thoughts of being dead or what it would be like to be dead	X	X	X	
I feel life is not worth living	X	X	X	X
Thoughts of hurting myself	X	X	X	X
Thoughts about ending my life	X	X	X	X
Thoughts that no one would care if I lived or died	X	X	X	X
Thoughts of harming self to feel better	X	X	X	X
Thoughts to end life but would not act	X	X	X	X
I have little doubt (am certain) about wanting to kill myself;	X	X	X	X
I have done something to hurt myself	X	X	X	
I cannot control my thoughts about killing myself	X	X	X	
I have started to work out the details to end my life	X	X	X	X
Worried about being judged socially if I hurt myself	X	X	X	
I have made preparations to kill myself	X	X	X	X
Hurt myself WITHOUT intent	X	X	X	X
Hurt myself WITH intent	X	X	X	X
I have hurt myself but I am not sure if I want to end my life	X	X	X	X
Attempt interrupted by others	X	X	X	X
I was about to do something to hurt/kill myself but stopped myself just before I initiated it	X	X	X	X
I planned to hurt or kill myself	X	X	X	
Can you rate the potential lethality of the behaviour?			X ^a^	

^a^ Item answered by clinicians only when there is a positive answer to a suicidal behaviour

- *STOP-SAS Children’s version*

The instrument for children comprised five items on suicidal ideation, two on suicide plans and three on suicidal behaviour. Two new items about suicidal behaviour were added to the children’s version: ‘aborted attempt’ and ‘undetermined suicide behaviour’, in order to cover the C-CASA algorithm. The children’s version thus had 12 items. Based on the advice of the STOP Scientific Advisory Board, we added two items capturing low-level suicidality for children, making the final version 14 questions long. Items included in the children’s version are shown in Table 1.

**Fig. 2:**
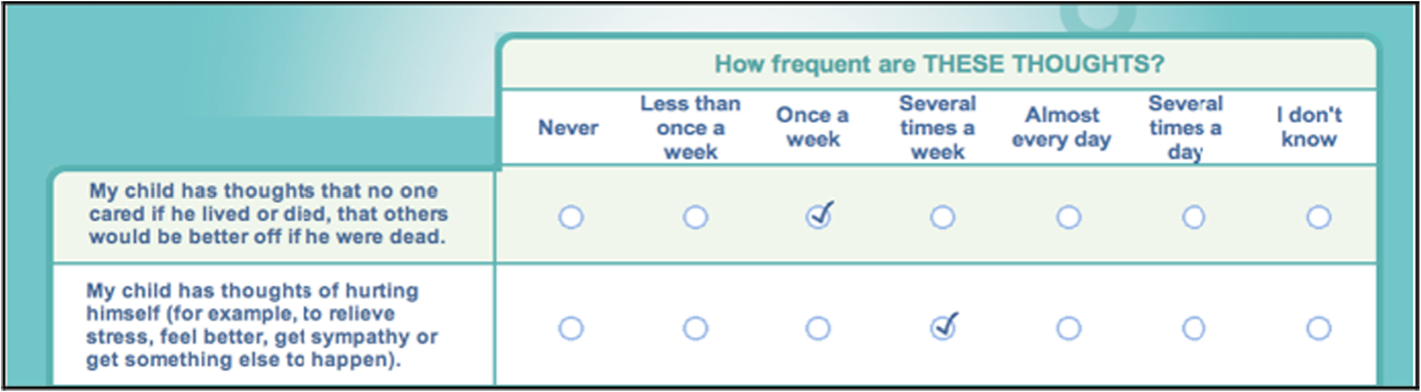
Example from the parent STOP-SAS questionnaire: statement, question and response options

An example of a couple of questions from the parent version of the STOP-SAS is shown in Fig. 2.

### Psychometric properties of the STOP-SAS questionnaires

- *Adolescent’, Parent’ and Clinician’ questionnaires*

A total of 395 adolescents, 637 parents completed the questionnaires, together with 716 questionnaires completed by clinicians. The adolescent and parent sample (*n* = 576), were all recruited from child and adolescent psychiatric outpatient settings in Spain (*n* = 191, 33.2%), United Kingdom (*n* = 155, 26.9%), Italy (*n* = 103, 17.9%), the Netherlands (*n* = 45, 7.8%), France (*n* = 45, 7.8%), and Germany (*n* = 37, 6.4%). Females comprised 51.4% of the sample, and the mean age was 14.94 ± 1.65 years. The main diagnoses and treatments are shown in Table 2.

**Table 2:** Clinical and demographic characteristics of participants in the validation study

Participants	Adolescents *n* = 576 (%)^a^	Children *n* = 187 (%)^a^
Diagnoses DSM-IV-TR*		
Eating disorder	65 (6.1)	0
Major Depressive Disorder (MDD)	258 (24)	16 (3.9)
Attention Deficit Hyperactivity Disorder (ADHD)	153 (14.3)	127 (30.6)
Conduct Disorder/Oppositional Defiant Disorder (CD/ODD)	89 (8.3)	60 (14.5)
Bipolar Disorder	39 (3.6)	6 (1.4)
Schizophrenia and Other Psychotic Disorders	36 (3.4)	0
Anxiety Disorders	129 (12)	35 (8.4)
Pervasive Developmental Disorders	114 (10.6)	67 (16.1)
Tic Disorders	25 (2.3)	13 (3.1)
Substance Related Disorders	14 (1.3)	0
Mental Retardation	55 (5.1)	35 (8.4)
Learning Disorders	20 (1.9)	12 (2.9)
Other Disorders	76 (7.1)	44 (10.6)
Pharmacological treatment		
Antipsychotics	367 (41)	143 (48)
Antidepressants	265 (29.6)	22 (7.4)
Stimulants	60 (6.7)	68 (22.8)
Mood stabilizers and Lithium	46 (5.1)	10 (3.4)
Benzodiazepines	36 (4)	1 (0.3)
Other psychiatric pharmacological treatments	56 (6.3)	35 (11.7)
Non-psychiatric pharmacological treatments	64 (7.1)	19 (6.6)
No pharmacological treatment	68 (11.08)^b^	19 (10.16)^b^

* DSM-IV-TR = The Diagnostic and Statistical Manual of Mental Disorders. Fourth edition, text revised

^a^ Percentage considering the total number of medications and diagnoses

^b^ Number of patients and percentage considering total number of patients

The STOP-SAS for adolescents, parents and clinicians showed adequate internal consistency. The best cross-informant correlation was found between clinicians and adolescents, although the correlation between parents and adolescents, and parents and clinicians was also high. Data regarding internal consistency and correlation between informants are shown in Table 3.

**Table 3:** Internal consistency and Cross-informant correlation coefficients

Psychometric properties: Internal consistency.		Cross-Informant correlation coefficients (Pearson’s r)		
	Cronbach’s alpha	PARENTS	ADOLESCENTS	CHILDREN
CLINICIANS	0.955	*r* = 0.591	*r* = 0.765	*r* = 0.640
(*n* = 716)		*p* < 0.001	*p* < 0.001	*p* < 0.001
ADOLESCENTS	0.964	*r* = 0.560		
(*n* = 395)		*p* < 0.001		
CHILDREN	0.922	*r* = 0.473		
(*n* = 110)		*p* < 0.001		
PARENTS	0.951			
(*n* = 637)				

In the one-way between-groups multivariate analysis of variance, the mean total score (range 0 to 100) for adolescents, parents and clinicians was the dependent variable. The independent variable was the version of the questionnaire. Results showed a significant difference between groups in the mean total score (*F*_2,322_ = 51.689.079, *p* < 0.001). It also showed a pattern in which adolescents presented with the highest score, parents the lowest score and clinicians in-between. Post-hoc pairwise comparisons between adolescents and parents and adolescents and clinicians were then performed (Bonferroni adjusted *p* value = 0.016). The mean total score of the STOP-SAS for adolescents (16.13 ± 19.93) was significantly higher than the total score for parents’ reports (7.01 ± 10.67; *t* =10.14 *p* < 0.001). It was also higher (17.44 ± 20.33) than the total score for clinicians’ report (11.52 ± 14.66; *t* = −8.72 *p* < 0.001). A strong correlation was found between the STOP-SAS total score for clinicians and the clinician-rated C-SSRS (*r* = 0.863, *p* < 0.001). The correlation with the C-SSRS was also strong for the adolescents’ SAS version (*r* = 0.670, *p* < 0.001) and the parents’ version (*r* = 0.548, *p* < 0.001). Regarding results from the ROC analysis, the accuracy of the clinicians’ version to identify suicidal ideation and behaviours (suicidality) was excellent compared with the C-SSRS. The accuracy of the adolescents’ version was good, and fair for the parents’ version. Results from the ROC analysis are showed in Table 4.

**Table 4:** Receiver Operating Characteristics between the STOP-SAS and the C-SSRS

	STOP-SAS cut off scores	Sensitivity (%)	Specificity (%)	ROC Area	SE	Asymptotic Normal (95% CI)	
Adolescents’ version	≥4.78	80.5	78.8	0.834	0.022	0.791	0.878
Children’s version	≥0.52	60	71.2	0.683	0.056	0.574	0.792
Parents’ version	≥0.43	72.7	69.8	0.756	0.020	0.716	0.796
Clinicians’ version	≥0.43	88.5	85.4	0.917	0.012	0.893	0.940

*ROC* Receiver Operating Characteristics, *SE* Standard Error, *CI* Confidential Interval

- *Children’s questionnaire*

A total of 110 children of 187 being seen in centres in Spain (*n* = 58, 31%), United Kingdom (*n* = 42, 22.5%), France (*n* = 33, 17.6%), Italy (*n* = 27, 14.4%), the Netherlands (*n* = 24, 12.8%) and Germany (*n* = 3, 1.6%) answered the questionnaires. Only 21.9% of the subjects were female, and the mean age was 9.86 ± 1.12 years. Clinical and treatment characteristics of the sample are shown in Table 2. The STOP-SAS for children showed good internal consistency. The correlation between informants was good between clinicians and children and between parents and clinicians but lower between parents and children (Table 3).

In the one-way between-groups multivariate analysis of variance, no significant differences were observed in the mean total scores (range 0–100) between children, parents and clinicians (*F*_2,102_ = 0.367, *p* = 0.694). A strong correlation was found between the STOP-SAS total score for children and the clinician-rated C-SSRS scores (*r* = 0.654, *p* < 0.001). Results for the children’s version regarding the ROC analysis against the C-SSRS were fair (Table 4).

### Screening questions

The correlation between the two variables created by adding up the screening questions and the rest of the questions, was very high for the four screening questions (*r* = 0.906, *p* < 0.001) and the three screening questions for children (*r* = 0.891, *p* < 0.001). For the two dichotomous variables created (Screening-questions and the STOP-SAS-RestQ) for adolescents, parents and adolescents together, the Kappa value was 0.843. For the same variables for children, the Kappa value was 0.723.

The overall estimation of agreement between the Screening-Questions and the rest of the STOP-SAS scale (the STOP-SAS-RestQ) showed that in 96.4% of the cases, if an adolescent, parent or clinician answered “never” to all the screening questions they also answered “never” to all the remaining questions. There was some inconsistency between the screening questions and the rest of the scale in seven adolescents, but after checking their specific answers and a discussion with the experts’ panel, they were classified as non-suicidal. The estimation of agreement between the three screening questions for children and the rest of the questionnaire showed similar results with only two cases that showed inconsistency. In this case reviewing their answers showed they were not suicidal. Data are shown in Table 5.

**Table 5:** Screening questions compared to the rest of the STOP-SAS questionnaire

Adolescents	Screening-questions = 0	Screening-questions = 1	total
The STOP-SAS-RestQ = 0	*n* = 185 (96.4%)	*n* = 20	*n* = 205
The STOP-SAS-RestQ = 1	*n* = 7	*n* = 137	*n* = 144
total	*n* = 192	*n* = 157	*n* = 349
Children			total
The STOP-SAS-RestQ = 0	*n* = 39 (95.1%)	*n* = 3	*n* = 42
The STOP-SAS-RestQ =1	*n* = 2	*n* = 9	*n* = 11
total	*n* = 41	*n* = 12	*n* = 53

## Discussion

The present study describes the development and validation of the STOP-SAS, a web-based PROM measuring suicidality on the HealthTracker^TM^ system using the FDA recommendations for outcome measure development. These initial psychometric validation data suggest that it is a reliable and valid instrument for assessing suicidality in children and adolescents. It also shows in all its versions, a significant correlation with the clinician-rated C-SSRS.

Adolescents in the focus groups clearly expressed the view that it was important to ask about suicidality. Suicide prevention is important in adolescents [25] and screening for suicidality in high school has been shown to be a safe component of youth suicide prevention programs [26]. It is worth noting that the adolescents said that they preferred a web-based questionnaire to a printed version, as they would be able to discuss their internal experiences more openly. Previous studies have reported that the use of computers increase the sense of privacy and may yield answers that are less socially desirable but more sincere [27, 28].

Due to development-related needs, the number of response options in the children’s questionnaire was reduced to four. Previous studies have recommended the use of between three and five responses for children between 8 and 11 years [29]. The recall period for children was modified from “over the last month” to “over the last few days”, because shorter recall periods and “here-and-now” type questions are preferable for this age group [30].

Psychometrically, internal consistency was good for all versions of the STOP-SAS. Agreement between informants (adolescents, parents and clinicians) regarding adolescents was good. This result contrasts with those of some studies that have reported low concordance between parents and adolescents when interviewed about psychopathology or suicidal ideation [31–33]. However, one previous study showed that the agreement between adolescents and parents was higher concerning recent suicide attempts than concerning attempts in the past [34]. Nevertheless, the mean total STOP-SAS score for adolescents was significantly higher than for parents. These findings corroborate those of previous studies that have reported under-recognition of suicidal ideation and other internalizing symptoms by parents compared to adolescents [31, 33, 35]. On the other hand, clinicians’ mean total scores were in-between parents’ and adolescents’ scores. These results suggest that adolescents disclose more easily information regarding suicidality to clinicians rather than to their parents.

Agreement between clinicians and children and between clinicians and parents was good, but lower between parents and children. This latter finding is consistent with previous research that has assessed psychopathology and parent and child agreement and has reported low cross-informant correlations, especially for internalizing symptoms [36–38]. No differences were found between the mean total scores of children, parents and clinicians.

There was a strong correlation between the clinician-rated C-SRSS total score and the clinicians’, parents’, adolescents’, and children’s STOP-SAS total scores. Moreover, results from the ROC analysis were excellent for the clinician version and good for adolescent and parent versions compared with the gold standard (C-SSRS). However, results were only fair for the children’s version. These findings suggest that the STOP-SAS is a valid instrument for assessing suicidality in adolescents, using self-reports or proxy reports by parents and clinicians. The interpretation of the results for the children’s version must be more cautious as it is possible that some children who are suicidal fail to report it, due to recall bias related to their memory capacity [38]. Other possible explanations for these results include the child misunderstanding the question or not feeling comfortable disclosing this information to a stranger. Nevertheless, some authors have suggested that all sources of information regarding suicidality in children should be considered when exploring child suicidality, as it is not clear who is the best reporter of suicidality. As parents may not detect suicidality in all cases and screening children may be necessary [38].

There is no data presented in this study on a test-retest of the measure. The test-retest reliability method was decided against, because we expect changes in suicidality even during short time-windows. The scores of items in the STOP-SAS would change with change in suicidality and hence test-retest was deemed inappropriate. It has been previously reported that for remitting and relapsing or episodic diseases, test retest reliability may be difficult or impossible to establish [12]. Previous studies with children and adolescents have found low test-retest reliability for scales measuring psychiatric symptoms or suicidality [39, 40]. This could be explained by the fact that symptoms such as suicidality and depression are expected to change over time, notably especially in young children [41]. In addition, in middle childhood (7–12 years old), memory capacity is still developing and retrospective questions may be a problem as children are prone to construct scripts of familiar routine if they do not remember the event [42].

One limitation of the development of the questionnaires is that focus groups were only held in Spanish, and the translations into English, French, Italian, Dutch and German were examined only by experts. Another limitation is that test-retest reliability has not been included as part of the psychometric properties of the new instruments due to the fact that suicidality is expected to change with time, especially in young children [41]. Another limitation is the sample size used for the validation of the children’s version that is quite small compared to the other groups, and that this version is less accurate in identifying suicidal ideation and behaviour (suicidality) compared with the C-SSRS. More reliable and valid results would be generated by including a larger sample of children with diverse degrees of suicidality; however the frequency of suicidal ideation or behaviour in children is very low and, therefore, it is difficult to recruit a sample with diverse degrees of suicidality. Given the population, one must acknowledge that external validity cannot be assumed across the adolescent population, but it does offer insight into the complex child and adolescent psychiatric population where suicidality is arguably more likely.

The STOP-SAS has a number of major strengths. Its development complies with the recommendations of the FDA for PROMs [12]; it is based on consultations with expert panels and the study of focus groups; it is available in different languages; and it obtains data from different sources, the patient, the parent and the clinician. On the advice of the Scientific Advisory Board, the STOP-SAS includes some questions about suicidal ideation assessing low-level suicidality such as “I have thoughts about being dead or what it would be like to be dead.”, “I feel life is not worth living.” “I have thoughts that no one cares if I lived or died, that others would be better off if I were dead.” Some authors have suggested that the C-SSRS is incomplete in assessing passive suicidal ideation [43, 44] and it may be potentially dangerous to ignore this type of thoughts. Further, the correlations with the clinician-rated Columbia-SSRS are strong. In addition, the STOP-SAS is available on the web-based HealthTracker^TM^ system, allowing scoring in real-time and providing an automated alerting system for suicide prevention programmes and pharmacovigilance. Moreover, the STOP-SAS includes questions about thoughts of self-harm that are not part of the C-SSRS. It could be argued that it may be important to ask about these thoughts for clinical practice and prevention of self-harm and acts of suicidal behaviours [44]. In addition, the questionnaire includes questions that measure the schema developed by Silverman [19] which differentiates between the presence or absence of suicidal intent, and between the presence or absence of injury, and adds a question to assess a third category “Undetermined Suicide-Related Behaviour: is a self-inflicted, potentially injurious behaviour where intent is unknown.

## Conclusions

In conclusion, the data shows that the STOP-SAS questionnaire is a comprehensive, rapid-to-use, web-based measure of suicidality for children and adolescents, using self and proxy measures from parents and clinicians. It has shown good internal consistency and good convergent validity with the C-SSRS. The children’s version shows good internal consistency and a good correlation with the C-SSRS; however the Area Under the Curve in the ROC analysis showed fair results when compared against the C-SSRS. An exploratory factor analysis and confirmatory factor analysis will now be performed to determine how many factors comprise the newly developed instruments. In addition, the STOP-SAS will be tested under the auspices of the STOP project in longitudinal paediatric observational cohorts involving children and adolescents taking atypical antipsychotics (risperidone, aripiprazole), children and adolescents with depression (treated with fluoxetine, non-pharmacological/cognitive behaviour therapy), children and adolescents with asthma or respiratory allergies (treated with montelukast or other medications) and a group of normal children and adolescents without psychopathology. Results from these trials will help to define how the HealthTracker^TM^-based STOP-SAS can best be used in registration trials, pharmacovigilance, and epidemiological and observational studies in the future.

## Abbreviations

C-CASA: Columbia classification algorithm of suicide assessment
C-SSRS: Columbia suicide severity rating scale
FDA: Food and Drug Administration
MRS: Medication-related suicidality
PCOMs: Patient centered outcome measures
ROC: Receiver operating characteristic
STOP: Suicidality: treatment occurring in pediatrics
STOP-SAS: STOP suicidality assessment scale
